# Blood metabolome shows signatures of metabolic dysregulation in obese and overweight subjects that can be predicted by machine learning applied to heart rate variability

**DOI:** 10.3389/fmolb.2025.1561987

**Published:** 2025-06-12

**Authors:** Andrea Di Credico, David Perpetuini, Pascal Izzicupo, Giulia Gaggi, Claudia Rossi, Arcangelo Merla, Barbara Ghinassi, Angela Di Baldassarre, Ines Bucci

**Affiliations:** ^1^ Department of Medicine and Aging Sciences, Chieti, Italy; ^2^ Center of Advanced Studies and Technologies (CAST), Chieti, Italy; ^3^ UdA-Tech Lab, G. D’Annunzio University of Chieti-Pescara, Chieti, Italy; ^4^ Department of Engineering and Geology, University “G. d’Annunzio” of Chieti-Pescara, Pescara, Italy; ^5^ Department of Science, “G. d’Annunzio” University of Chieti-Pescara, Chieti, Italy; ^6^ Department of Innovative Technologies in Medicine and Dentistry, “G. d’Annunzio” University Chieti-Pescara, Chieti, Italy

**Keywords:** obesity, metabolic disturbances, heart rate variability, amino acids, acylcarnitine

## Abstract

**Introduction:**

Obesity and overweight are linked to metabolic disturbances, which contribute to the onset of diseases like type 2 diabetes (T2D) and cardiovascular disorders. Metabolic health is also closely linked to autonomic function, as measured by heart rate variability (HRV), making HRV a potential non-invasive indicator of metabolic status. While studies have examined metabolic changes with body mass index (BMI), the link between HRV and specific metabolic profiles in normal-weight (NW), overweight (OW), and obese (OB) individuals is less understood. Additionally, whether HRV can reliably predict key metabolites associated with metabolic dysregulation remains largely unexplored.

**Methods:**

This study uses targeted metabolomics to profile amino acids and acylcarnitines in a group of academic employees across BMI categories (NW, OW, and OB) and investigates correlations between HRV variables and these metabolites. Finally, a machine learning approach was employed to predict relevant metabolite levels based on HRV features, aiming to validate HRV as a non-invasive predictor of metabolic health.

**Results:**

NW, OW, and OB subjects showed different metabolic profiles, as demonstrated by sparse partial least square discriminant analysis (sPLS-DA). The main upregulated metabolites differentiating NW from OB were C6DC and C8:1, while C6DC and C10:2 were higher in OW than NW. Time- and frequency-domain HRV features show a good correlation with the regulated metabolites. Finally, our machine learning approach allowed us to predict the most regulated metabolites in OB and OW subjects using HRV metrics.

**Conclusion:**

Our study advances our understanding of the metabolic and autonomic changes associated with obesity and suggests that HRV could serve as a practical tool for non-invasively monitoring metabolic health, potentially facilitating early intervention in individuals with elevated BMI.

## Introduction

Obesity and overweight have emerged as leading health concerns, posing significant challenges to healthcare systems worldwide (Hruby and Hu, 2015). These conditions are associated with a substantial increase in the risk of developing numerous chronic diseases, notably T2D and cardiovascular diseases (CVDs), which collectively contribute to a high percentage of global morbidity and mortality rates (Powell-Wiley et al., 2021).

At the molecular level, excess body fat is linked to profound metabolic alterations that disrupt lipid, amino acid, and energy metabolism. These alterations contribute to insulin resistance, chronic low-grade inflammation, dyslipidemia, and hypertension, which are major features of metabolic syndrome (Ruze et al., 2023). As the understanding of these metabolic disruptions has deepened, the importance of profiling specific metabolic pathways has gained prominence in the study of obesity.

The metabolomics-based approach provides an opportunity to analyze specific classes of metabolites, facilitating an in-depth assessment of biochemical variations (Danzi et al., 2023). Amino acids have been shown to have robust associations with obesity and insulin resistance, suggesting their critical role in metabolic health.

Among amino acids, both branched-chain amino acids (BCAAs) and aromatic amino acids (AAAs) have shown robust associations with obesity and insulin resistance, suggesting their critical roles in metabolic health (Sun et al., 2020). Notably, aromatic amino acids—including phenylalanine, tyrosine, and tryptophan—have been increasingly recognized for their role in metabolic dysregulation. Elevated levels of phenylalanine and tyrosine have been strongly associated with impaired glucose metabolism, systemic inflammation, and cardiovascular risk (Adams, 2011). Their accumulation may reflect dysfunction in the catabolism of amino acids and altered signaling pathways, making them highly relevant biomarkers for metabolic health assessment. Elevated levels of BCAAs and other essential amino acids have been identified in individuals with higher body mass index (BMI), often correlating with an increased risk of metabolic disorders (Adams, 2011; Yoon, 2016). These amino acids are implicated in insulin signaling pathways and are known to influence energy balance and lipid metabolism, potentially aggravating metabolic dysregulation.

Acylcarnitines, which facilitate the transport of fatty acids into mitochondria for beta-oxidation, are also increasingly recognized for their role in obesity-related metabolic dysfunction (Longo et al., 2016). When lipid oxidation becomes impaired, intermediate metabolites, including various acylcarnitine species, accumulate, reflecting mitochondrial stress and reduced metabolic flexibility (Kankuri et al., 2023).

Targeted metabolomics using tandem mass spectrometry allows the simultaneous determination of multiple metabolites. Notably, this analysis can be performed on capillary blood collected as dried blood spot (DBS) samples using a non-invasive and simple approach, which is suitable for large-scale studies.

The autonomic nervous system (ANS) plays a fundamental role in regulating metabolic processes, acting as a bridge between the central nervous system and peripheral organs such as the heart, pancreas, and adipose tissue (Imai and Katagiri, 2022). Through its sympathetic and parasympathetic branches, the ANS exerts rapid and adaptable control over key physiological functions, including glucose homeostasis, lipid mobilization, and thermogenesis (Ruud et al., 2017). In individuals with obesity or metabolic syndrome, the balance between sympathetic and parasympathetic activity is often disrupted, leading to a phenomenon known as sympathetic overactivity, which can further exacerbate metabolic dysfunction (Malpas, 2010).

Heart rate variability (HRV) serves as a practical, non-invasive marker for autonomic balance. By measuring the variations in the intervals between heartbeats, HRV reflects the dynamic interplay between sympathetic and parasympathetic inputs to the heart (Shaffer and Ginsberg, 2017; Di Credico et al., 2022; Di Credico et al., 2024a). A higher HRV generally indicates healthy autonomic function with a greater ability to adapt to physiological demands, while a lower HRV is often observed in conditions associated with metabolic dysfunction, such as obesity, diabetes, and CVD (Supriya et al., 2021; Di Credico et al., 2024b). Research has highlighted that reduced HRV is associated with insulin resistance, inflammation, and increased cardiovascular risk, underscoring the potential of HRV as an indicator of metabolic health (Zeid et al., 2024).

Given its ease of measurement and established link with autonomic regulation, HRV has gained attention as a potential tool for assessing metabolic health non-invasively. Studies have found significant correlations between HRV variables and specific metabolic markers, supporting the hypothesis that HRV could reflect underlying metabolic status (Azulay et al., 2022).

In this regard, targeted metabolomics using tandem mass spectrometry allows the simultaneous determination of multiple metabolites, including key aromatic amino acids and acylcarnitines, providing critical insights into metabolic dysfunctions associated with obesity. Notably, this analysis can be performed on capillary blood collected as DBS samples using a non-invasive and simple approach that is suitable for large-scale studies.

However, research exploring the relationship between HRV and targeted metabolite profiles across BMI categories remains limited.

The present study seeks to bridge this gap by examining the correlation between HRV variables and targeted metabolites (specifically amino acids and acylcarnitines, two classes of metabolites critically involved in energy balance, lipid metabolism, and cellular signaling pathways) in a sample of academic employees grouping them by BMI into normal-weight (NW), overweight (OW) and obese (OB) categories. Finally, we employ a machine learning (ML) approach to explore whether HRV variables can predict relevant metabolites. Indeed, predictive modeling offers the potential to use HRV as a non-invasive tool for estimating metabolic alterations, providing a cost-effective alternative for assessing metabolic health. By doing so, this study aims to explore whether HRV can serve as a surrogate marker for metabolic disturbances, providing clinicians with an accessible method to assess metabolic health in individuals at risk for metabolic disorders.

## Materials and methods

### Study design and ethical considerations

Twenty-eight subjects (14 men and 14 women) were included in the present cross-sectional study. For study purposes, participants were divided into three groups based on their BMI category: normal weight: NW (*n* = 15), overweight: OW (*n* = 7), and obese: OB (*n* = 6). After overnight fasting, capillary finger blood was collected on filter paper cards to obtain DBS samples. Briefly, after warming and disinfecting, the finger prick was done with a nearly painless device. The first drop of blood was removed by a sterile gauze pad, and then two or three drops of blood were soaked into the filter paper. Collection cards were left to dry horizontally for at least 3 h (Cicalini et al., 2022). On the same day, 5-min HRV and bioimpedance data were obtained at rest. Data collection took place in a controlled environment with no external noise, and the ambient temperature was maintained between 20°C and 25°C during all analyses to avoid environmental stressors that could affect cardiac autonomic responses and bioimpedance measurements. The study was approved by the Research Ethics Board of the University of Chieti-Pescara (approval number: 1479, date of approval: 03/05/2017), and it followed the principles of the Declaration of Helsinki. Each participant signed the informed consent, and participants could withdraw from the experiment at any time.

### Targeted metabolomics profiling and data analysis

Targeted metabolic profiling of DBS samples was performed by flow injection analysis-tandem mass spectrometry (FIA-MS/MS). DBS samples were punched out into 3.2-mm-diameter disks to extract a panel of metabolites, including amino acids, free carnitine and acylcarnitines, ketones, and nucleosides. Details of DBS sample preparation using NeoBaseTM 2 Non-derivatized MSMS kit (Revvity, Turku; Finland) have been already described. In particular, the procedure involves the extraction of analytes from the punched disks with a solution containing labeled internal standards and analysis using an MS/MS system (Cicalini et al., 2021; Rossi et al., 2020). The FIA-MS/MS system consisted of a RenataDX Screening System (Waters Corporation, Milford, MA, United States). The system operates in positive electrospray ionization mode by multiple reaction monitoring (MRM) acquisition. A 10-μL aliquot of each extracted sample was injected into the ion source, and the run time was 1.1 min, injection-to-injection. Processing of the data was carried out by MassLynxTM (IVD) Software V4.2 with IonLynxTM Application Manager (Waters Corp., Wilmslow, United Kingdom).

The metabolite concentration dataset was uploaded as a.csv file in MetaboAnalyst version 6.0. Metabolite concentration was normalized by the median before any further analysis. A spare partial least square-discriminant analysis (sPLS-DA) was implemented to classify the three groups based on the entire metabolite set. A list of the metabolites used in the present study is reported as Supplementary Material (Supplementary Table S1). Volcano plot analysis for the two-group analysis (i.e., NW vs. OW and NW vs. OB subjects) and the enrichment analysis and graphs were obtained using MetaboAnalyst, version 6.0.

### HRV measurement and analysis

The RR intervals were acquired using a Bodyguard 2 (Firstbeat Technologies Ltd., Jyväskylä, Finland) wearable device that was positioned according to the manufacturer’s instructions. Bodyguard 2 records the ECG signal with electrodes, processes the signal with an integrated algorithm, and provides beat-to-beat RR intervals as an output with a 1 ms resolution. Participants were instructed to avoid stimulant foods and drinks, including coffee, as well as strenuous physical exercise in the days leading up to the data acquisition day to minimize potential confounding factors. For female participants, HRV data collection was controlled for the menstrual cycle. All assessments were performed during the mid-follicular phase (between day +5 and +10, with the onset of menses considered day +1) when progesterone levels are low and stable, following established guidelines (Schmalenberger et al., 2020). The device end was attached to the right side of the body under the collarbone, whereas the cable end was attached to the left side of the body on the rib cage. A continuous 5-min HRV recording was used for analysis, following a standardized 10-min rest period to ensure that participants reached a homeostatic balance. The HRV data were analyzed using Kubios HRV Standard 3.4.0 software. All the signals were visually inspected for artifacts, and the threshold-based algorithm (low-threshold) available in Kubios software was applied if needed. This approach has been shown to have minimal impact on HRV data integrity, as low-level corrections are sufficient to remove artifacts without significantly altering the results (Cutrim et al., 2024). Regarding time-domain analysis, mean RR intervals, standard deviation of NN intervals (SDNN), root mean square of successive differences (RMSSD), and the stress index (i.e., the square root of Baevsky’s stress index) were obtained. Regarding the frequency domain, low-frequency (LF) and high-frequency (HF) data were obtained.

### Whole-body bioimpedance assessment

Bioimpedance analysis was performed using BIA (BIA 101 Anniversary AKERN s.r.l., Florence, Italy) with an electric current at a frequency of 50 kHz (±1%). The device was calibrated before assessment using the standard control circuit supplied by the manufacturer with a known impedance (resistance [R] = 380 Ω; reactance [Xc] = 45 Ω). The device’s accuracy was 0.1% for R and 0.1% for Xc. For the bioelectrical impedance measurement, each participant was positioned supine for a minimum of 2 min to distribute the body fluid evenly. During this time, the legs were positioned at 45° relative to the midline of the body, while the upper limbs were positioned 30° away from the trunk. After cleaning the skin with alcohol, two electrodes (Biatrodes, AKERN s.r.l., Florence, Italy) were placed on the back of the right hand and two electrodes on the neck of the corresponding foot (Di Credico et al., 2021a). Fat mass data were obtained using Bodygram software.

### Machine learning procedures

A multivariate regression analysis was conducted to predict the metabolite concentrations based on HRV parameters. While various ML methodologies may be appropriate for this objective, the restricted number of participants necessitated a simplified procedure; thus, a support vector regression (SVR) with a linear kernel was used to mitigate the risk of overfitting. Moreover, to further mitigate the potential overfitting resulting from an excessive number of features relative to the sample size, a feature selection technique (i.e., minimum-redundancy maximum-relevance method, MRMR) was implemented inside the cross-validation (CV) framework. The first six features were used for the regression of the metabolite concentrations. The study sample was randomly split into a train set (23 participants) and a test set (five participants). The performance of the model was tested to evaluate the correlation between the estimated and measured metabolite concentrations. The analysis was performed using MATLAB R2023b (MathWorks, Inc., Natick, MA, United States).

### Statistical analysis

Metabolites were considered up- or downregulated when the fold change between groups was >1.5 and *p* < 0.05. The Shapiro–Wilk test was run to check for data normality. When the normality was violated, a non-parametric test was selected. One-way analysis of variance (ANOVA) or Kruskal–Wallis was used to check for differences between the three groups. When a difference was found, Tukey’s *post hoc* test for multiple comparisons was used. Independent *t*-tests or Mann–Whitney tests were performed to check for differences in the two-group comparisons. Pearson’s r or Spearman rho was used to measure the correlation between metabolites and HRV metrics, and partial correlation was computed to check for the influence of sex and age. Correlation analysis was performed to evaluate the performance of the applied ML model and the regression equation, and *R* ^2^ values were obtained. Inferential statistics and related graphs were made using Prism 10.1.1 (GraphPad Software, LLC). All the results were considered statistically significant when *p* < 0.05.

## Results

### Participants’ characteristics

Table 1 summarizes the participant characteristics, grouping them by BMI. The study sample consisted of 28 participants (14 men, mean age 50.231 ± 6.784; 14 women, mean age 51.929 ± 8.435), further divided into 15 normal-weight, seven overweight, and six obese subjects considering BMI and body composition. No difference was found in the mean age, height, and resting minimum heart rate between the three groups. Conversely, weight, BMI, fat mass percentage, and high frequency (HF peak) of HRV were significantly different. All participants did not report any acute cardiovascular conditions, and they were non-smokers in apparently stable health.

**TABLE 1:** Participant characteristics based on the BMI category.

Variables	NW	OW	OB	*p*
*n*	15	7	6	
Age (years)	50.267 ± 9.067	53.143 ± 6.986	52.500 ± 4.461	0.684
Weight (kg)	61.180 ± 6.446	78.886 ± 10.701	96.600 ± 16.617	**<0.001**
Height (cm)	165.267 ± 6.867	168.686 ± 10.453	168.717 ± 10.710	0.588
BMI	22.351 ± 1.200	27.653 ± 1.493	34.002 ± 5.455	**<0.001**
Fat mass (%)	24.607 ± 6.017	33.650 ± 3.829	35.650 ± 8.127	**0.002**
Minimum heart rate (bpm)	67.511 ± 8.583	75.337 ± 8.380	70.583 ± 11.675	0.209
Mean RR (ms)	806.242 ± 95.028	740.167 ± 76.159	788.610 ± 113.513	0.338
RMSSD (ms)	36.805 ± 15.205	26.663 ± 3.026	26.390 ± 3.746	0.059
SDNN (ms)	33.863 ± 11.335	25.354 ± 5.904	28.062 ± 7.733	0.132
Stress index	12.623 ± 3.031	16.472 ± 4.796	15.721 ± 4.038	0.069
HF peak (Hz)	0.293 ± 0.082	0.276 ± 0.094	0.182 ± 0.023	**0.046**
LF peak (Hz)	0.083 ± 0.034	0.083 ± 0.036	0.208 ± 0.292	0.575

ANOVA or Kruskal–Wallis (for weight, BMI, RMSSD, SDNN, HF peak, and LF peak) were performed, and *p*-values were reported. Results are reported as mean ± standard deviation, and they were considered significant when *p* < 0.05. Significant p-values are highlighted in bold.

NW, normal weight; OW, overweight; OB, obese.

### Dried blood spot metabolomic profile of the three subgroups

**FIGURE 1:**
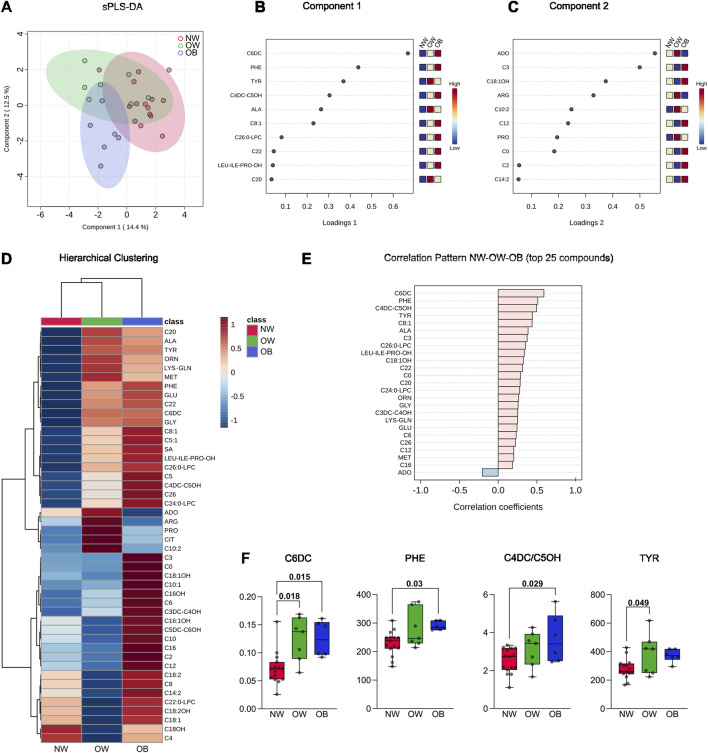
**(A)** Spare partial least square-discriminant analysis (sPLS-DA) of blood metabolites from the 15 normal-weight, seven overweight, and six obese subjects involved in the study. The 2D plot reports the sample projection of the first and second components of the sPLS-DA. **(B)** Metabolite loadings of principal component 1. **(C)** Metabolite loadings of principal component 2. **(D)** The hierarchical clustering graph reports the difference in metabolite abundance in the three different groups analyzed and their clustering; the whole panel of 46 metabolites detected is included in the heatmap. Both dendrogram (to show groups and metabolite clustering) and heatmap (to show metabolite abundance) are reported. **(E)** Correlation analysis was performed in MetaboAnalyst using data from NW, OW, and OB as continuous (i.e., BMI); the top 25 correlated metabolites are reported. **(F)** Boxplots show the concentration of the significantly different metabolites. The Kruskal–Wallis test was used for C6DC, while one-way analysis of variance (ANOVA) was used for PHE, C4DC/C5OH, and TYR. Single data points and adjusted *p*-values are shown.

When comparing the fasting plasma metabolome profiles of OB and OW with NW, a clear separation of the two groups from NW was observed in the spare partial least square-discriminant analysis (sPLS-DA), and this was especially evident between OB (blue dots) and NW (red dots) subjects (Figure 1A). Components 1 and 2 together explained 26.9% of the variation (component 1 = 14.4%; component 2 = 12.5%) (Figure 1A). The loading plots show that the three metabolites most involved in the variance in component 1 were adipylcarnitine (C6DC), phenylalanine (PHE), and tyrosine (TYR) (Figure 1B). On the other hand, the first three metabolites most responsible for the group classification in component 2 were adenosine (ADO), propionylcarnitine (C3), and 3-hydroxy-octadecenoylcarnitine (C18:1OH) (Figure 1C).

Hierarchical clustering was performed to obtain a preliminary view of how the metabolites were regulated in the three groups. Considering the entire metabolite dataset, the hierarchical clustering dendrogram for samples grouped NW and OW classes, while the OB class was the most divergent (Figure 1D), thus confirming the visual net separation demonstrated by the sPLS-DA. Indeed, the heatmap showed that at least 33 of 46 metabolites were more abundant in the OB group than in the NW group, while their concentration was more similar between NW and OW (Figure 1D).

The correlation pattern analysis of the 25 most correlated compounds showed that the concentration of a major part of metabolites was positively correlated with BMI. Specifically, C6DC, PHE, methylmalonylcarnitine/3-hydroxy-valerylcarnitine (C4DC/C5OH), and TYR show a Pearson’s correlation coefficient of 0.5 and higher, while ADO showed an opposite trend, being slightly negatively correlated with BMI (Figure 1E).

Considering the most correlated metabolites, inferential statistics were performed to assess differences between the three subgroups. One-way ANOVA showed that C6DC, PHE, C4DC/C5OH, and TYR metabolites were significantly different between groups. Tukey’s *post hoc* test demonstrated that C6DC was higher in OW and OB than NW. Differently, PHE and C4DC/C5OH were only significantly higher in OB than in NW. Finally, TYR showed higher values in OW subjects than the NW counterpart (Figure 1F).

### Differentially regulated metabolites in NW versus OB, NW versus OW, and enrichment analysis

**FIGURE 2:**
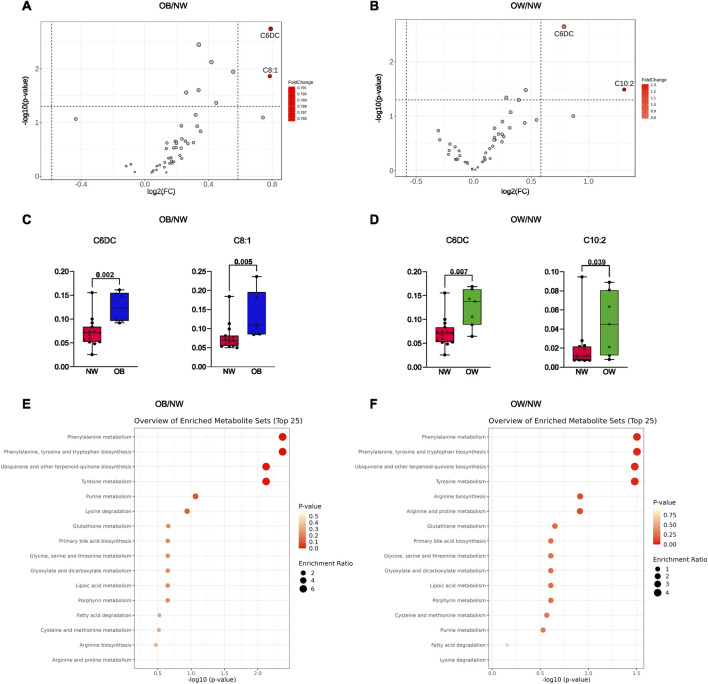
**(A)** Volcano plot representing the comparison of the metabolic profiles between OB and NW. **(B)** Volcano plot representing the comparison of the metabolic profiles between OW and NW. Metabolites highlighted in red were considered upregulated. **(C)** Histograms refer to independent t-tests for the metabolites with different abundances between OB and NW. **(D)** Histograms refer to Mann–Whitney tests for the metabolites with different abundances between OW and NW. Adjusted *p*-values are shown. **(E)** Enrichment analysis for metabolic pathways using the KEGG database comparing OB and NW subgroups. **(F)** Enrichment analysis for metabolic pathways using the KEGG database comparing OW and NW subgroups.

For the two-group comparison analysis, volcano plots were created to check for differently regulated metabolites between OB/NW and OW/NW groups (settings were FC >1.5 and *p* < 0.05). Results showed that compared to NW, in the OB subgroup, C6DC and octenoylcarnitine (C8:1) were significantly more abundant (Figures 2A,C). Similarly, C6DC and decadienoylcarnitine (C10:2) were also significantly more abundant in the OW than in the NW subjects (Figures 2B,D). Interestingly, C6DC was found to be upregulated in both the OB and OW subgroups compared to NW (Figures 2A,B).

Afterward, an enrichment analysis was performed to explore the profile of functionally relevant metabolites and determine a link between changes in metabolite expression and the biological meaning. Interestingly, for both enrichment analyses (OB vs. NW and OW vs. NW), results showed the highest enrichment of metabolites involved in “phenylalanine metabolism,” “phenylalanine, tyrosine and tryptophan biosynthesis,” “ubiquinone and other terpenoid-quinone biosynthesis,” and “tyrosine metabolism,” suggesting alterations in these metabolic processes (Figures 2E,F).

The data collectively indicate significant metabolic differences between NW, OW, and OB individuals. Specific metabolites, such as C6DC and C8:1, showed significant differences between these groups. The enrichment analyses suggest that pathways involved in amino acid metabolism, quinone biosynthesis, and other metabolic processes are altered in individuals with overweight and obesity.

### Correlation between metabolites and HRV features

**FIGURE 3:**
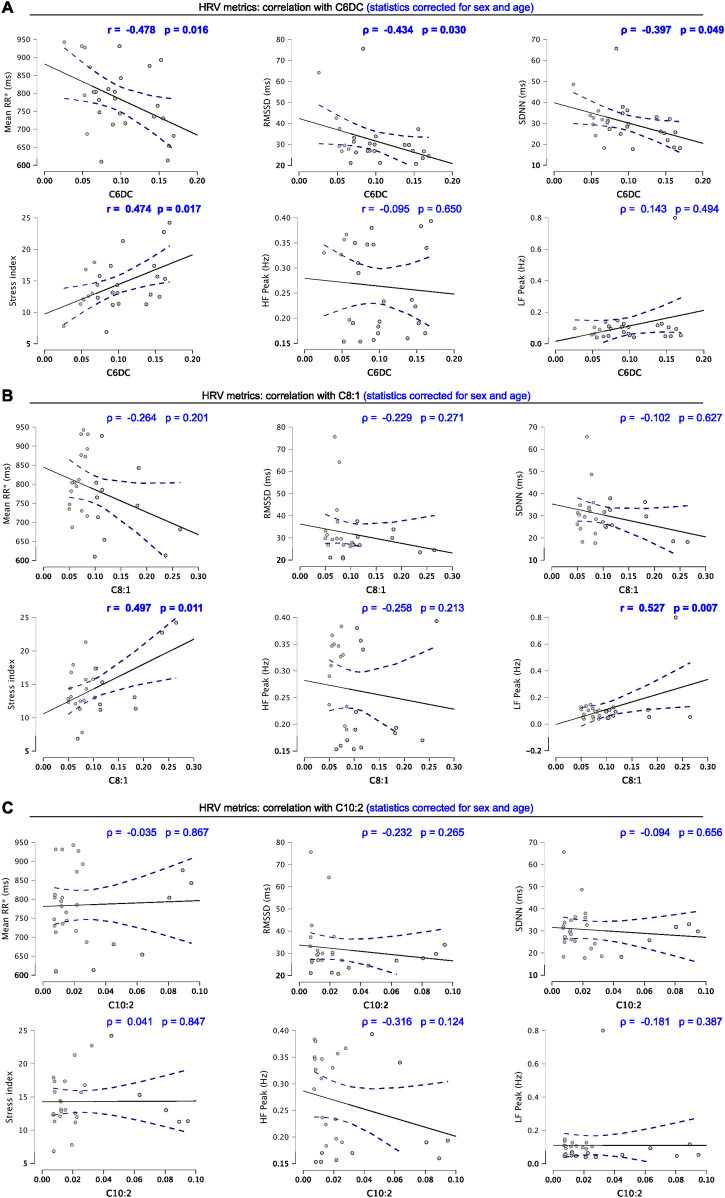
**(A)** Correlation graphs of C6DC and HRV metrics. **(B)** Correlation graphs of C8:1 and HRV metrics. **(C)** Correlation graphs of C10:2 and HRV metrics. Scatter plots include Pearson’s r or Spearman’s ρ and level of significance corrected for age and sex. Significant correlations are highlighted (bold). Blue dotted lines represent 95% confidence intervals. Results were considered significant when *p* < 0.05.

To investigate the relationship between metabolic state and autonomic state tuning, the upregulated metabolites found in the two-group comparison (C6DC, C8:1, and C10:2) were used to compute correlation analysis with clinical-relevant time and frequency-domain HRV metrics (mean RR intervals; RMSSD, SDNN, stress index, HF peak, and LF peak). Correlations were also adjusted for sex and age to check for their influence. Considering adjusted values, significant moderate negative correlations were observed between C6DC and mean RR interval (*r* = −0.478, *p* = 0.016). RMSSD and SDNN were also negatively correlated (*ρ* = − 0.434, *p* = 0.030, *ρ* = − 0.397, *p* = 0.049). Conversely, a positive correlation was observed between C6DC and stress index (*r* = 0.474, *p* = 0.017) (Figure 3A).

Similarly, regarding the metabolite C8:1, a positive correlation was found with the stress index (*r* = 0.497, *p* = 0.011). C8:1 was also positively correlated with the frequency-domain LF peak (*r* = 0.527, *p* = 0.007) (Figure 3B). Finally, no significant correlation was noted between C10:2 and the selected HRV metrics, demonstrating that among the upregulated metabolites in overweight and obese individuals, C10:2 is less related to autonomic cardiac regulation (Figure 3C).

### Prediction of relevant metabolites through ML applied to HRV data

**FIGURE 4:**
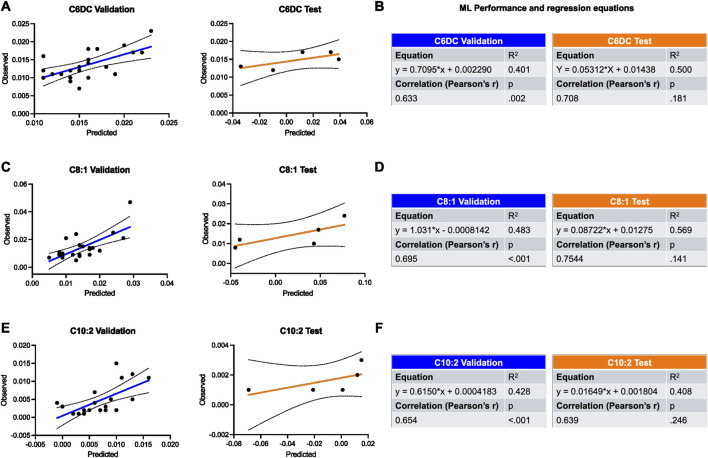
Validation and test of the applied ML model for the prediction of most regulated metabolites in OW and OB groups using HRV features. **(A,B)** Prediction of C6DC is shown by plots and tables containing the results of regression and correlation. **(C,D)** Prediction of C8:1 is shown by plots and tables containing the results of regression and correlation. **(E,F)** Prediction of C10:2 is shown by plots and tables containing the results of regression and correlation. Black dotted lines represent 95% confidence intervals. Results were considered significant when *p* < 0.05.

The HRV features extracted were used to train an ML model to predict the most regulated metabolites in our OB and OW samples, namely, C6DC (Figures 4A,B), C8:1 (Figures 4C,D), and C10:2 (Figures 4E,F). The ML validation was performed on data from 23 individuals, and the data from five were used for the test. For both validation and test, the prediction performance was evaluated using Pearson’s correlation coefficient and calculating the *R* ^2^ as well.

Regarding the validation, ML showed a good performance in all cases, and the highest was for the prediction of C8:1 (*R* ^2^ = 0.483, *r* = 0.695, *p* = 0.002). Similarly, the best prediction for the test set was seen for C8:1 as well (*R* ^2^ = 0.569, *r* = 0.754, *p* = 0.141) (Figures 4C,D).

## Discussion

This study provides novel insights into the metabolic and autonomic alterations associated with different BMI categories, highlighting significant changes in the metabolic profile of overweight and obese. Using targeted metabolomics, we identified key metabolites—such as adipylcarnitine (C6DC) and octenoylcarnitine (C8:1)—that were upregulated in individuals with higher BMI, reflecting metabolic inflexibility and mitochondrial stress. Correlation analysis revealed significant associations between HRV features and these metabolite levels, underscoring the interplay between autonomic regulation and metabolic health. Notably, we employed an ML approach to predict metabolite concentrations based on HRV metrics, demonstrating that HRV could serve as a non-invasive biomarker for metabolic disturbances. These findings reinforce the potential role of HRV analysis in metabolic health monitoring and support its application in early detection strategies for individuals at risk of obesity-related metabolic dysfunction.

Our metabolomics findings, notably the upregulation of C6DC, PHE, TYR, and C8:1 in overweight and obese subjects, align with previous research identifying distinct amino acid and acylcarnitine profiles in obesity. Elevated amino acid levels, including phenylalanine and tyrosine, have been consistently associated with insulin resistance, inflammatory states, and increased cardiovascular risk. Increases in PHE and TYR levels in individuals with obesity reflect this, suggesting a shift toward amino acid metabolism dysregulation that may contribute to obesity-related pathologies (White and Newgard, 2019). BCAAs and AAAs are known to impair insulin signaling and mitochondrial function when elevated, fostering an environment conducive to metabolic syndrome and T2D (Lynch and Adams, 2014).

The acylcarnitines, particularly the elevated C6DC and C8:1, indicate impaired fatty acid oxidation, reflecting mitochondrial stress and reduced metabolic flexibility in the overweight and obese groups (McCann et al., 2021). This corroborates prior work showing that specific acylcarnitines accumulate under conditions of metabolic inflexibility, a hallmark of obesity and associated metabolic disorders (Mihalik et al., 2010). These patterns support the view that obesity profoundly disrupts both amino acid and fatty acid metabolism, with implications for the progression of insulin resistance, dyslipidemia, and other metabolic diseases.

The enrichment of phenylalanine and tyrosine metabolism pathways underscores the established link between aromatic amino acids and obesity-related metabolic dysfunction. Elevated levels of aromatic amino acids, including phenylalanine and tyrosine, are commonly observed in individuals with higher BMI and have been implicated in insulin resistance and systemic inflammation (Würtz et al., 2013). These amino acids are known to influence insulin signaling and lipid metabolism, and their dysregulation may contribute to metabolic disturbances that characterize obesity and metabolic syndrome.

The identified enrichment in tryptophan biosynthesis and tyrosine metabolism is also notable as these pathways are precursors to key neurotransmitters, such as serotonin and dopamine, which influence appetite regulation, energy balance, and mood. Altered tryptophan and tyrosine pathways could contribute to changes in neurotransmitter availability, potentially impacting the central mechanisms that regulate energy intake and expenditure (Hildebrand et al., 2015). Additionally, impaired neurotransmitter biosynthesis may affect mood and reward-driven eating behaviors, which are known to be altered in individuals with obesity (Labban et al., 2020). This link between neurotransmitter biosynthesis and obesity suggests that metabolic alterations in aromatic amino acids may extend beyond purely physiological effects, influencing behavioral and psychological factors associated with weight gain and obesity.

The enrichment in ubiquinone and other terpenoid–quinone biosynthesis pathways highlights a potential link between obesity and mitochondrial function. Ubiquinone plays a critical role in mitochondrial electron transport and oxidative phosphorylation, processes essential for ATP production (Deshwal et al., 2023). Disruptions in ubiquinone biosynthesis, which may lead to reduced availability of coenzyme Q, could impair mitochondrial efficiency and contribute to the observed metabolic inflexibility in obesity (Wang and Hekimi, 2013). This impaired energy production aligns with our findings of elevated acylcarnitines, which are markers of incomplete fatty acid oxidation and mitochondrial stress (Bouchouirab et al., 2018). Taken together, these observations support the hypothesis that obesity-related mitochondrial dysfunction, in part due to altered ubiquinone biosynthesis, contributes to the metabolic rigidity and energy imbalance observed in individuals with elevated BMI.

The observed pathway enrichments provide important insights into the metabolic shifts associated with obesity and highlight specific biochemical pathways that may serve as therapeutic targets. Addressing the disruptions in amino acid metabolism and mitochondrial function could help alleviate some of the metabolic challenges faced by individuals with obesity, potentially mitigating the risk of developing metabolic syndrome, insulin resistance, and related disorders. These enrichment findings emphasize the need for further research into interventions that target these pathways, aiming to restore metabolic flexibility and improve overall metabolic health in individuals at risk.

Our study further elucidates the relationship between HRV and metabolic health by revealing moderate negative correlations between HRV metrics (e.g., mean RR interval, SDNN, and RMSSD) and certain metabolites, particularly C6DC. Reduced HRV metrics, such as SDNN and mean RR interval, are associated with increased sympathetic dominance, which is common in obesity and metabolic syndrome (Ortiz-Guzmán et al., 2023; Di Credico et al., 2024c). The positive correlation between C6DC and stress index, as well as C8:1 and LF peak, further highlights the potential of HRV as an indicator of metabolic dysfunction. These findings align with those of research indicating that obesity-induced autonomic dysregulation may contribute to metabolic alterations (Shibao et al., 2007), as sympathetic overactivity in obesity influences glucose and lipid metabolism, increasing cardiovascular and metabolic risks (Kalil and Haynes, 2012).

Despite the growing evidence for a potential surrogate for metabolic health, our findings suggest that only select metabolites (such as C6DC and C8:1) showed significant correlations with HRV metrics, underscoring that HRV might selectively reflect aspects of metabolic status, particularly those linked with sympathetic dominance and mitochondrial function. This supports HRV as a valuable, yet partial, marker for broader metabolic disturbances associated with elevated BMI.

The use of ML to predict metabolite levels from HRV variables demonstrates an innovative approach for estimating metabolic health in overweight and obese. Our model yielded good predictive performance, particularly for C8:1, supporting the feasibility of using HRV features to estimate metabolite concentrations. This approach aligns with previous studies demonstrating the utility of machine learning in predicting valuable physiological metrics (Di Credico et al., 2021b; Perpetuini et al., 2021; Perpetuini et al., 2023) and indicates HRV as a potential predictive marker for early metabolic dysregulation.

This model’s predictive accuracy for metabolites as C8:1 may indicate that certain acylcarnitines, potentially those linked with mitochondrial β-oxidation, have a closer association with autonomic markers than amino acids. The high predictive performance for C8:1 suggests that HRV could be further optimized to identify specific metabolites of interest in obesity and related metabolic conditions.

From a clinical standpoint, identifying early alterations in C8:1 levels could serve as a biomarker for subclinical metabolic dysfunction before overt symptoms or disease manifest. The ability to predict such alterations through HRV may offer a practical approach for early metabolic screening in at-risk populations. This presents an opportunity for HRV-based tools that could enable non-invasive assessment of specific metabolic alterations in clinical practice, offering a potential approach for early risk assessment and metabolic monitoring in populations at risk for obesity-related disorders.

### Study implications and future directions

The findings of this study have several implications. First, the distinct metabolomic profiles in overweight and obese subjects reinforce the importance of metabolic monitoring as a means to understand disease risk progression. Second, HRV correlations with key metabolites and their predictive power via machine learning suggest that HRV could serve as a useful, non-invasive marker for metabolic health, potentially guiding early interventions. Of note, HRV and the metabolic profile obtained by FIA-MS/MS on DBS samples represent an approach suitable for larger-scale studies. Future studies should consider expanding on these findings by including larger and more diverse cohorts to validate HRV as a predictive marker for metabolomic alterations. Additionally, research into the longitudinal relationship between HRV and metabolic profiles could shed light on the potential for HRV to serve as an early indicator of metabolic dysregulation before the onset of overt disease. Machine learning approaches could also be further refined to enhance the predictive accuracy of HRV-derived estimates of metabolite concentrations, potentially leading to practical applications in healthcare settings where early, non-invasive screening methods for metabolic dysfunction are needed (Romero-Saldaña et al., 2018).

### Limitations

This study has certain limitations. For this reason, it could be considered a preliminary research work, and the results could be expanded in future studies. The relatively small sample size may limit the generalizability of the findings, and future studies with larger and more diverse populations are needed to validate our results. Additionally, while BMI was used to classify participants, other clinical parameters related to obesity, such as lipid profiles, insulin resistance, and inflammatory markers, were not available, which might have provided further insights into the metabolic state of the participants. Another limitation is the cross-sectional design, which precludes establishing causal relationships between autonomic regulation and metabolic alterations. Despite controlling for key factors such as sex and age, residual confounding variables cannot be entirely ruled out. Finally, while our ML approach demonstrated promising predictive capabilities, the limited sample size may have constrained the complexity and robustness of the model. Nevertheless, despite these limitations, this study offers valuable insights into the relationship between HRV and metabolomic profiles across BMI categories. The integration of targeted metabolomics, HRV analysis, and ML highlights the potential for non-invasive tools to assess metabolic health, paving the way for future research and clinical applications. The rigorous methodology, including strict cross-validation, enhances the reliability of our findings, and our work contributes to the growing field of metabolic research, emphasizing the importance of autonomic regulation in metabolic health assessment.

## Conclusion

In conclusion, this study contributes to our understanding of the metabolic and autonomic changes associated with increased BMI and highlights the potential of HRV as a surrogate marker for metabolic health. The integration of HRV analysis with metabolomics and machine learning represents a promising avenue for non-invasive assessment and personalized health monitoring in obesity and related metabolic disorders. Future research should aim to build on these findings to develop clinically applicable tools for early detection and intervention in metabolic dysregulation, ultimately improving patient outcomes and reducing the healthcare burden associated with obesity-related diseases.

## Data Availability

The raw data supporting the conclusions of this article will be made available by the authors, without undue reservation.
