# Supplementary material for: Blood metabolome shows signatures of metabolic dysregulation in obese and overweight subjects that can be predicted by machine learning applied to heart rate variability
Source: Front Mol Biosci. 2025 Jun 12;12:1561987. doi: 10.3389/fmolb.2025.1561987 (PMC12197924; doi:10.3389/fmolb.2025.1561987)
Supplement: Supplementary file 1 [file DataSheet1.pdf]

Electrospray ionization tandem mass spectrometry (ESI-MS/MS) acquisition parameters for the analysis of whole blood amino acids (AAs) and acylcarnitines (ACs). MS/MS transitions for each analysed AA and AC, the optimal cone potential (V), and collision energy (eV) are shown for each analyte. The capillary potential was 3.5 kV.

Abbreviation	Full name	Transition	Cone potential	Collision energy
**ALA**	Alanine	90.2>44.3	40	6
**ARG**	Arginine	175.3>70.3	45	17
**CIT**	Citrulline	176.2>113.2	45	13
**LYS-GLN**	Lysine/Glutamine	147.2>130.2	40	12
**GLU**	Glutamic Acid	148.2>84.2	40	8
**GLY**	Glycine	76.2>30.3	40	5
**LEU-ILE-PRO-OH**	Leucine/Isoleucine/Hydroxyprolin	132.2>86.3	40	8
**MET**	Methionine	150.2>104.2	45	9
**ORN**	Ornithine	133.3>70.3	40	12
**PHE**	Phenylalanine	166.2>120.2	45	11
**PRO**	Proline	116.1>70.1	28	12
**SA**	Succynilacetone	155.1>109.1	24	22
**TYR**	Tyrosine	182.2>136.2	45	12
**ADO**	Adenosine	168.1>136.1	32	18
**C0**	Free Carnitine	162.2>103.2	60	14
**C10:2**	Decadienoylcarnitine	313.>85.2	75	19
**C2**	Acetylcarnitine	204.2>85.2	60	14
**C3**	Propionylcarnitine	218.2>85.2	60	15
**C3DC-C4OH**	Malonylcarnitine/3-Hydroxy-butyrylcarnitine	248.3>85.2	60	15
**C4**	Butyrylcarnitine	232.3>85.2	60	15
**C4DC-C5OH**	Methylmalonylcarnitine/3-Hydroxy-valerylcarnitine	262.2>85.2	70	16
**C5**	Valerylcarnitine	246.2>85.2	70	16
**C5:1**	Tiglylcarnitine	244.2>85.2	70	16
**C5DC-C6OH**	Glutarylcarnitine/3-Hydroxy-hexanoylcarnitine	276.3>85.2	70	20
**C6**	Hexanoylcarnitine	260.3>85.2	65	16
**C6DC**	Adipylcarnitine	282.3>85.2	70	20
**C8**	Octanoylcarnitine	288.3>85.2	75	18
**C8:1**	Octenoylcarnitine	286.3>85.2	75	18
**C10**	Decanoylcarnitine	316.3>85.2	75	19
**C10:1**	Decenoylcarnitine	314.3>85.2	75	19
**C12**	Dodecenoylcarnitine	344.4>85.2	75	22
**C14:2**	Tetradecadienoylcarnitine	368.4>85.2	75	23
**C16**	Hexadecanoylcarnitine (palmitoylcarnitine)	400.4>85.2	75	25
**C16:1OH**	3-Hydroxy-hexadecenoylcarnitine	414.4>85.2	75	25
**C16OH**	3-Hydroxy-hexadecanoylcarnitine	416.4>85.2	75	25
**C18:1**	Octadecenoylcarnitine (Oleylcarnitine)	426.4>85.2	80	25
**C18:1OH**	3-Hydroxy-octadecenoylcarnitine	442.4>85.2	80	25

Abbreviation	Full name	Transition	Cone potential	Collision energy
**C18:2**	Octadecadienoylcarnitine (Linoleylcarnitine)	424.4>85.2	80	25
**C18:2OH**	3-Hydroxy-octadecadienoylcarnitine	440.3>85.0	56	28
**C18OH**	3-Hydroxy-octadecanoylcarnitine	444.4>85.2	80	25
**C20**	Eicosanoylcarnitine (Arachidoylcarnitine)	456.4>85.0	69	34
**C22**	Docosanoylcarnitine (Behenoylcarnitine)	484.4>85.0	69	34
**C26**	Hexacosanoylcarnitine (Cerotoylcarnitine)	540.5>85.0	69	34
**C20:0-LPC**	Tetracosanoylcarnitine (Lignoceroylcarnitine)	552.4>104.1	74	30
**C24:0-LPC**	C24:0 lysophosphatidylcholine	608.5>104.1	74	30
**C26:0-LPC**	C26:0 lysophosphatidylcholine	636.5>104.1	74	30
